# Baltic Salmon, *Salmo salar*, from Swedish River Lule Älv Is More Resistant to Furunculosis Compared to Rainbow Trout

**DOI:** 10.1371/journal.pone.0029571

**Published:** 2012-01-20

**Authors:** Lars Holten-Andersen, Inger Dalsgaard, Kurt Buchmann

**Affiliations:** 1 Department of Veterinary Disease Biology, Faculty of Life Sciences, University of Copenhagen, Frederiksberg C, Denmark; 2 Division of Veterinary Diagnostics and Research, National Veterinary Institute, Technical University of Denmark, Copenhagen, Denmark; Universitat de Barcelona, Spain

## Abstract

**Background:**

Furunculosis, caused by *Aeromonas salmonicida*, continues to be a major health problem for the growing salmonid aquaculture. Despite effective vaccination programs regular outbreaks occur at the fish farms calling for repeated antibiotic treatment. We hypothesized that a difference in natural susceptibility to this disease might exist between Baltic salmon and the widely used rainbow trout.

**Study Design:**

A cohabitation challenge model was applied to investigate the relative susceptibility to infection with *A. salmonicida* in rainbow trout and Baltic salmon. The course of infection was monitored daily over a 30-day period post challenge and the results were summarized in mortality curves.

**Results:**

*A. salmonicida* was recovered from mortalities during the entire test period. At day 30 the survival was 6.2% and 34.0% for rainbow trout and Baltic salmon, respectively. Significant differences in susceptibility to *A. salmonicida* were demonstrated between the two salmonids and hazard ratio estimation between rainbow trout and Baltic salmon showed a 3.36 higher risk of dying from the infection in the former.

**Conclusion:**

The finding that Baltic salmon carries a high level of natural resistance to furunculosis might raise new possibilities for salmonid aquaculture in terms of minimizing disease outbreaks and the use of antibiotics.

## Introduction

The bacterial disease furunculosis caused by *Aeromonas salmonicida* is one of the main concerns in European salmonid mariculture due to high mortality rates and significant economic losses [1], [2]. Vaccination programs have kept the problem under some control. However, side effects following oil-adjuvanted i.p. vaccination have raised a series of ethical and welfare questions related to the use of vaccines [3], [4], [5]. Hence, inherent resistance in the fish against furunculosis would be preferable in order to reduce medication and side effects from immunoprophylactic procedures. Recent studies have shown that the isolated salmon stock in the Baltic possesses genes conferring resistance towards the extremely pathogenic parasite *Gyrodactylus salaris* [6], [7], [8]. This salmon stock comprises numerous sub-populations homing to rivers in Sweden, Finland, Russia, Latvia, Lithuania, Estonia, Poland and Germany draining into the Baltic Sea [9]. Baltic salmon from rivers Ume älv and Lule älv present a clear protective immune response a few weeks after infection with *G. salaris* [8], [10], [11], [12]. In contrast, East-Atlantic salmon (Norwegian, Scottish, and Danish) are very susceptible to *G. salaris* and show no effective immune response during infection [6], [7], [8], [10], [13]. These differences between salmon stocks regarding protective immunity against the very pathogenic *G. salaris* pose the question whether a comparable difference might exist when it comes to infection with the bacterium *A. salmonicida*. Positive correlation between resistance to furunculosis and infectious salmon anaemia (viral) in farmed Atlantic salmon have previously been reported [14]. On the other hand, a successful breeding program for increased resistance in brook trout (*Salvelinus fontinalis*) to furunculosis also led to higher susceptibilities to *Gyrodactylus* sp., bacterial gill disease, and *Chilodonella* sp. infections [15]. Although several studies have investigated the potential difference between various salmonids with regard to inherent resistance to *A. salmonicida* [16], [17], [18] a comparison between Baltic salmon and rainbow trout has not previously been carried out. In the present study, a population of East-Atlantic salmon naturally infected with *A. salmonicida* was used as disease carriers in a cohabitation study to test for differences in susceptibility to furunculosis between Baltic salmon and rainbow trout. Here, we present evidence that the Baltic salmon stock compared to rainbow trout carries a high level of natural resistance against *A. salmonicida*. Further, the study confirmed a previously reported high susceptibility in East-Atlantic salmon.

## Results

### Development of furunculosis in East-Atlantic salmon

**Figure 1:**
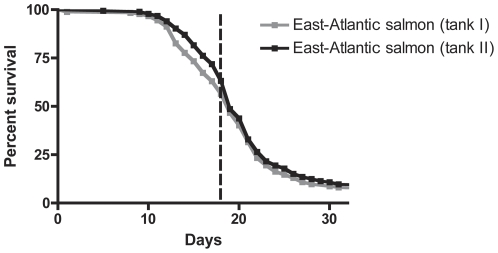
Mortality curves for naturally infected East-Atlantic Salmon. The data summarizes mortality in 300 salmon from duplicate tanks each with 150 fish/tank. The stippled line shows the time-point for randomly picking batches of infected East-Atlantic salmon as cohabitants from parallel tanks (cohab-tanks) with comparable mortalities.

The course of furunculosis in the group of East-Atlantic salmon started as a low-grade infection that developed into wide spread disease with a significant increase in mortalities from day 10 (Fig. 1). At day 30 the numbers of fish alive in the two replicate tanks were 14 and 15, respectively (9.7% in total). Bacteria isolated from the kidney of dead fish were identified as *A. salmonicida* subsp. *salmonicida*.

### Difference between Baltic salmon and rainbow trout in natural resistance against *A. salmonicida*

**Figure 2:**
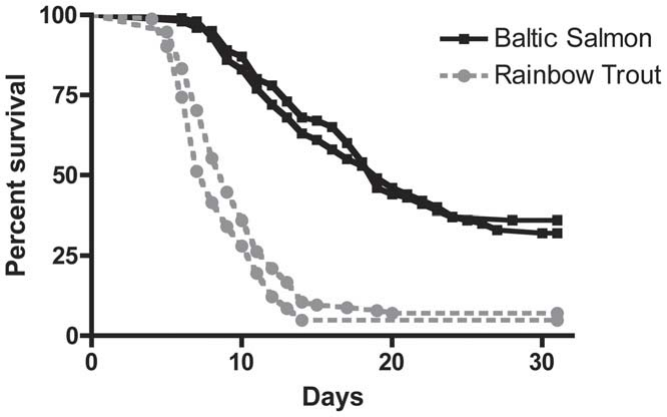
Mortality curves for Baltic salmon and rainbow trout infected through cohabitation (day 0) with *A. salmonicida* infected East-Atlantic Salmon. Curves in black and grey color summarize mortality in 200 fish from duplicate tanks with 100 Baltic salmon or rainbow trout per tank. Non-infected control groups showed zero mortality over the course of the experiment.

The cohabitation infection model proved to be effective in terms of disease transmission. At day four and six post exposure (transfer of 50 infected East-Atlantic salmon) mortality was recorded in rainbow trout and Baltic salmon, respectively (Fig. 2). Bacteriological examination confirmed that mortalities resulted from infection with biochemically identical *A. salmonicida*, thus verifying transmission of disease from East-Atlantic salmon to Baltic salmon and rainbow trout.

The median survival time for Baltic salmon and rainbow trout was 19 and eight days, respectively (Table 1). A chi-square test for independence demonstrated a significant difference between the survival curves of Baltic salmon and rainbow trout (Table 1). Calculating the hazard ratio showed that the relative risk of dying from infection with *A. salmonicida* is 3.36 times higher in rainbow trout compared to Baltic salmon.

**Table 1:** Descriptive statistics for differences in resistance to *A. salmonicida* infection in Baltic salmon and rainbow trout.

	Median survival	Chi-square	Hazard ratio^a^
Baltic salmon	19 days	128.5 (P<0.0001)	3.36 (4.08–6.94)
Rainbow trout	8 days		

a Hazard ratio with 95% confidence interval.

Linear regression was performed for the linear sections of the mortality curves representing the three salmonids. The analysis generated three slopes that are presented in Table 2.

**Table 2:** Slope and 30 day survival in East-Atlantic salmon, Baltic salmon and rainbow trout.

	East-Atlantic salmon	Baltic salmon	Rainbow trout
Slope^a^	1.5	0.75	2.4
30 day survival	9.7%	34.0%	6.2%

a Slope estimated by linear regression analysis for the linear sections of the mortality curves in Fig. 1 and Fig. 2.

Rainbow trout showed the steepest decline (highest mortality rate) followed by East-Atlantic salmon and Baltic salmon, in that order. In concordance with these results the fraction of survivors (30 day survival) in the three salmonid species was 6.2% in rainbow trout, 9.7% in East-Atlantic salmon, and 34.0% in Baltic salmon (Table 2). No mortality was found in any control group.

## Discussion

Susceptibility to *A. salmonicida* infection was in a direct comparison demonstrated to differ between Baltic salmon and rainbow trout. A significantly higher survival (34%) was found in the Baltic salmon populations over a 30-day infection course compared to rainbow trout (6.2%). A chi-square test and hazard ratio estimation between rainbow trout and Baltic salmon confirmed their difference in susceptibility. East-Atlantic salmon could not be included in these estimations due to their status as a naturally infected population and hence their role as infected cohabitants. That means, the East-Atlantic salmon group should be considered a 100% infected population as opposed to the 33% infection level in the cohabitant groups at the beginning of the experiment. This difference might have affected the kinetics of the infections. Nonetheless, the 30-day survival data (Table 2) indicated a clear trend for a higher susceptibility of East-Atlantic salmon compared to Baltic salmon. Rainbow trout is normally considered more resistant to furunculosis compared to other salmonids (e.g. Atlantic salmon, brown trout, and brook trout) [18], [19], [20], [21]. However, the susceptibility of salmon stocks from the Baltic to *A. salmonicida* have not previously been tested and the present study is the first to compare resistance to furunculosis in Baltic salmon and rainbow trout.

The mechanisms responsible for the observed difference in resistance between the three salmonids were not investigated in this study. Yet, there are several reports describing factors that might influence resistance to infection in salmonid species. As a first line of defense natural barriers of the skin and the mucus with anti-bacterial properties have been suggested to play a major role [17], [20], [22]. In the present study, the later onset of mortalities (and confirmed disease) in the Baltic salmon compared to rainbow trout could indicate that the Baltic salmon carries a more resistant exterior as described above. However, a likely entry route besides skin and gills for *A. salmonicida* to the fish is crossing the intestinal lining [23] and systemic disease was confirmed in mortalities of all three salmonids. Thus, the higher survival at the end of the test period in the Baltic salmon compared to both rainbow trout and East-Atlantic salmon points to other defense mechanisms in addition to external barriers. It suggests that means to control systemic disease are present in the Baltic salmon. In this regard, both innate and adaptive anti-bacterial mechanisms are likely to be involved. Moreover, the extreme polymorphisms found at some major histocompatibility complex (MHC) loci and the existence of high- and low-resistance MHC alleles in Atlantic salmon with regard to *A. salmonicida* [24], [25], [26], [27] calls for outbred populations when testing for inherent disease resistance in a given species. Hence, it should be stressed that the population of Baltic salmon used for the present study originated from eggs collected from 100 female fish and fertilized with individual males to eliminate or heavily reduce the effect of individual family differences. As part of the innate response a range of circulating proteins act through neutralizing bacteria or activate downstream effecter mechanisms [28], [29], [30], [31]. During early stages of an *A. salmonicida* infection Atlantic salmon react with a strong and specific humoral response, which during chronic infection is substituted for a less effective response dominated by unspecific natural antibodies [32]. Additionally, opsonization followed by phagocytic clearance [33], [34], natural antitoxins [35], [36], [37], and production of immune complexes [38] are all described as essential parts of an effective anti-bacterial defense in salmonids. Whether these mechanisms or other elements are the reason for the observed survival in the Baltic salmon remains to be investigated.

Differential susceptibility to other pathogens between Baltic salmon, rainbow trout and East-Atlantic salmon has been reported previously. A well-described example is the clear difference in susceptibility between these salmonids to infections with the pathogenic ectoparasitic monogenean *G. salaris* [6], [7], [10], [11], [39]. An additional report presented a difference in susceptibility between these fish to infestations with another monogenean species, *Gyrodactylus derjavinoides* [40]. The exact mechanisms responsible for these differences have only been partly elucidated but seem to include variations in expression patterns of a series of cytokine and effector molecules [7], [39]. Susceptibility to bacterial kidney disease (BKD) caused by the gram-positive bacterium *Renibacterium salmoninarum* also differs significantly among salmonids, with Pacific salmon species being the most susceptible and rainbow trout the least [41], [42]. Baltic salmon were not included in these studies. A comparison between Atlantic salmon and several *Oncorhynchus* spp. in regard to their relative resistance to infectious salmon anaemia (ISA) showed a significantly higher susceptibility and mortality in the Atlantic salmon [43]. Additionally, heritability estimates of susceptibility among Chinook salmon (*Oncorhynchus tshawytscha*) and Atlantic salmon indicated that the heritability component is more pronounced for BKD than for some other bacterial diseases, including furunculosis [16], [44].

In salmonid aquaculture the infection pressure with *A. salmonicida* can periodically be substantial [1]. To prevent furunculosis, caused by this bacterium, fish farmers vaccinate their fish and use antibiotics in case of disease outbreak. However, currently used vaccines may cause problematic side effects in the fish [3], [4], [5]. Moreover, reducing antibiotic treatment remains a goal of aquaculture producers in order to avoid the outlet of antimicrobial residues and the development of resistance in the bacteria [45], [46]. In this light, the increased resistance of Baltic salmon to furunculosis shown in the present study may have a series of important implications for future salmon farming since inherent resistance to pathogens could be a means to reduce the need for medication. Moreover, vaccination studies showed that a single vaccination of Baltic salmon smolt eliminated mortality during a four month net-pen period and increased recapture rates significantly after stocking [47], [48]. Hence, the combination of improved vaccines with diminutive side effects and use of disease resistant fish stock in the production line may further reduce the need for medication in mariculture. In this regard, the possible use of at least some sub-populations of Baltic salmon should be further investigated. However, the choice of species can obviously not rely solely on one parameter, e.g. resistance to *A. salmonicida*, but needs to take into consideration the differences in susceptibilities to other pathogens in addition to level of domestication, feed conversion rate, and growth potential [49] of the individual species.

## Materials and Methods

### Ethics statement

The Committee for Animal Experimentation, Ministry of Justice, Copenhagen, Denmark, approved the study including the fish rearing and experimentation (license number 2006/561-1204), which was performed following the ethical guidelines listed in the license.

### Fish and rearing conditions

Baltic salmon eggs were collected from 100 wild female spawners and fertilized with sperm from individual wild males all of certified stocks of Baltic salmon (*Salmo salar*, River Lule älv, Vattenfall AB, Umeå, Sweden). The river Lule älv strain is considered an original Baltic salmon strain kept isolated from East-Atlantic salmon stocks for thousands of years [9]. Rainbow trout eggs were obtained from Fousing Trout Farm, Jutland, Denmark. The eggs were collected from more than 50 female spawners (Fousing strain) and fertilized with sperm from six males. To secure a high diversity in the population the egg pool were mixed following incubation. Salmon and rainbow trout eggs were brought to the hatchery and disinfected using iodophore (Actomar K30). Subsequently, they were hatched and fish reared under pathogen-free conditions for three months in recirculated water (Bornholm Salmon Hatchery, Denmark). Hereafter, the fish were brought to our experimental fish keeping facility. The pathogen-free status of the fish was tested and confirmed before the experiment was initiated. In addition, East-Atlantic salmon (River Skjern å, Denmark) carrying a natural infection with *A. salmonicida* were brought to our facility from a salmon hatchery in Jutland, Denmark, for use as infected cohabitants. These fish came from an egg pool based on four female and three male East-Atlantic salmon from River Skjern å, Denmark. The River Skjern å salmon strain is considered to be an original ancient Danish stock, which is currently used for re-stocking of rivers in western Denmark. All fish were acclimated for two weeks and kept in 200 L tanks (200 fish/tank) with bio-filters (Eheim, Germany). Fish were maintained at a 12 h light and 12 h dark cycle in aerated (100% oxygen saturation) tap water at 13°C. All fish were selected for similarity in size (weight 4–5 g). In addition to the above mentioned permission granted by the Committee for Animal Experimentation, Ministry of Justice, Copenhagen, Denmark (see ethics statement), the fish rearing was approved as part of the current restocking program for Baltic salmon in River Lule älv (no file number).

### Bacteria

*A. salmonicida* was isolated from the natural infected East-Atlantic salmon in October 2010, and the infected fish was used for the cohabitation challenge experiment. Isolation was performed on blood agar (blood agar base CM55, Oxoid, supplemented with 5% citrated calf blood) at 20°C for 48 h and the bacteria was identified by the following criteria ([50]): haemolysis, pigment production, cytochrome oxidase, motility, degradation of glucose, arginine dihydrolase, lysine and ornithine decarboxylase, indole and aesculin. Dead and moribund fish were examined bacteriologically to confirm cause of death.

### Experimental design

Six groups were established with duplicate tanks for each group: a) Baltic salmon control (no infection; 150 fish/tank), b) rainbow trout control (no infection; 150 fish/tank), c) Baltic salmon+rainbow trout control (no infection; 75+75 fish/tank), d) East-Atlantic salmon (infected, 150 fish/tank), e) Baltic salmon+infected East-Atlantic salmon (100+50 fish/tank), f) rainbow trout+infected East-Atlantic salmon (100+50 fish/tank).

### Infection procedure

Fish (Baltic salmon and rainbow trout) were infected through cohabitation with *A. salmonicida*-carrying East-Atlantic salmon. Infected salmon used for cohabitation were tagged (fin-clipped) in order to differentiate these from Baltic salmon and rainbow trout. The use of infected fish as cohabitants provided a natural disease transmission. Initially, disease development and mortality in the infected East-Atlantic salmon was recorded. In addition to the duplicate tanks described above for this group (d) two additional tanks (cohab-tanks) each holding 200 naturally infected East-Atlantic salmon were set up to produce the cohabitants for effective disease transmission. Dead fish were removed and counted on a daily basis from group (d) during course of infection. When a stable infection was established (Fig. 1) batches of 50 fish were randomly picked from the parallel cohab-tanks and transferred to groups (e) and (f) for infection of Baltic salmon and rainbow trout, respectively. This time-point was day 0 for group (a), (b), (c), (e) and (f). Again, dead fish were removed and counted on a daily basis.

### Statistics

The Prism© software package (version 4.0 for Macintosh, GraphPad Software, Inc.) was used to manage data and for statistical analyses. Death from infection was summarized in mortality curves and slopes at the linear section of each curve were estimated by linear regression analysis for comparison between East-Atlantic salmon, Baltic salmon and rainbow trout. The chi-square test for independence was used to test for difference in survival between Baltic salmon and rainbow trout. The hazard ratio (here describing the relative risk of dying from infection) between Baltic salmon and rainbow trout is presented with the 95% confidence interval (CI) [51]. The significance level was set at 0.05.

## References

[1] Pedersen K, Skall HF, Lassen-Nielsen AM, Nielsen TF, Henriksen NH (2008). Surveillance of health status on eight marine rainbow trout, Oncorhynchus mykiss (Walbaum), farms in Denmark in 2006.. J Fish Dis.

[2] Press CM, Lillehaug A (1995). Vaccination in European salmonid aquaculture: a review of practices and prospects.. Br Vet J.

[3] Berg A, Bergh Ø, Fjelldal PG, Hansen T, Juell JE (2006). Animal welfare and fish vaccination - effects and side-effects.. Fisken og Havet.

[4] Koppang EO, Bjerkas I, Haugarvoll E, Chan EK, Szabo NJ (2008). Vaccination-induced systemic autoimmunity in farmed Atlantic salmon.. J Immunol.

[5] Koppang EO, Haugarvoll E, Hordvik I, Aune L, Poppe TT (2005). Vaccine-associated granulomatous inflammation and melanin accumulation in Atlantic salmon, Salmo salar L., white muscle.. J Fish Dis.

[6] Bakke TA, Jansen PA, Hansen LP (1990). Differences in host resistance of Atlantic salmon, Salmo salar L., stocks to the monogenean Gyrodactylus salaris Malmberg, 1957.. J Fish Biol.

[7] Kania PW, Evensen O, Larsen TB, Buchmann K (2010). Molecular and immunohistochemical studies on epidermal responses in Atlantic salmon Salmo salar L. induced by Gyrodactylus salaris Malmberg, 1957.. J Helminthol.

[8] Lindenstrøm T, Sigh J, Dalgaard MB, Buchmann K (2006). Skin expression of IL-1beta in East Atlantic salmon, Salmo salar L., highly susceptible to Gyrodactylus salaris infection is enhanced compared to a low susceptibility Baltic stock.. J Fish Dis.

[9] Nilsson J, Gross R, Asplund T, Dove O, Jansson H (2001). Matrilinear phylogeography of Atlantic salmon (Salmo salar L.) in Europe and postglacial colonization of the Baltic Sea area.. Mol Ecol.

[10] Dalgaard MB, Nielsen CV, Buchmann K (2003). Comparative susceptibility of two races of Salmo salar (Baltic Lule river and Atlantic Conon river strains) to infection with Gyrodactylus salaris.. Dis Aquat Organ.

[11] Heinecke RD, Martinussen T, Buchmann K (2007). Microhabitat selection of Gyrodactylus salaris Malmberg on different salmonids.. J Fish Dis.

[12] Kania P, Larsen TB, Ingerslev HC, Buchmann K (2007). Baltic salmon activates immune relevant genes in fin tissue when responding to Gyrodactylus salaris infection.. Dis Aquat Organ.

[13] Bakke TA, MacKenzie K (1993). Comparative susceptibility of native Scottish and Norwegian stocks of Atlantic salmon, Salmo salar, to Gyrodactylus salaris Malmberg: Laboratory experiments.. Fish Res.

[14] Ødegård J, Olesen I, Gjerde B, Klemetsdal G (2007). Positive genetic correlation between resistance to bacterial (furunculosis) and viral (infectious salmon anaemia) diseases in farmed Atlantic salmon (Salmo salar).. Aquaculture.

[15] Hayford CO, Embody GC (1930). Further progress in the selective breeding of brook trout at the New Jersey State Hatchery.. Trans Am Fish Soc.

[16] Beacham TD, Evelyn TPT (1992). Genetic-Variation in Disease Resistance and Growth of Chinook, Coho, and Chum Salmon with Respect to Vibriosis, Furunculosis, and Bacterial Kidney-Disease.. Trans Am Fish Soc.

[17] Cipriano RC, Ford LA, Jones TE (1994). Relationship between resistance of salmonids to furunculosis and recovery of Aeromonas salmonicida from external mucus.. J Wildl Dis.

[18] Ellis AE, Stapleton KJ (1988). Differential susceptibility of salmonid fishes to furunculosis correlates with differential serum enhancement of Aeromonas salmonicida extracellular protease activity.. Microb Pathog.

[19] Cipriano RC (1982). Furunculosis in brook trout: infection by contact exposure.. Prog Fish Cult.

[20] Cipriano RC, Heartwell CM (1986). Susceptibility of Salmonids to Furunculosis - Differences between Serum and Mucus Responses against Aeromonas-Salmonicida.. Transactions of the American Fisheries Society.

[21] Mccarthy DH (1983). An Experimental-Model for Fish Furunculosis Caused by Aeromonas-Salmonicida.. J Fish Dis.

[22] Hjelmeland K, Christie M, Raa J (1983). Skin Mucus Protease from Rainbow-Trout, Salmo-Gairdneri Richardson, and Its Biological Significance.. J Fish Biol.

[23] Ringø E, Jutfelt F, Kanapathippillai P, Bakken Y, Sundell K (2004). Damaging effect of the fish pathogen Aeromonas salmonicida ssp. salmonicida on intestinal enterocytes of Atlantic salmon (Salmo salar L.).. Cell Tissue Res.

[24] Kjøglum S, Larsen S, Bakke HG, Grimholt U (2008). The effect of specific MHC class I and class II combinations on resistance to furunculosis in Atlantic salmon (Salmo salar).. Scand J Immunol.

[25] Grimholt U, Larsen S, Nordmo R, Midtlyng P, Kjøglum S (2003). MHC polymorphism and disease resistance in Atlantic salmon (Salmo salar); facing pathogens with single expressed major histocompatibility class I and class II loci.. Immunogenetics.

[26] Lohm J, Grahn M, Langefors A, Andersen Ø, Storset A (2002). Experimental evidence for major histocompatibility complex-allele-specific resistance to a bacterial infection.. Proc Biol Sci.

[27] Langefors A, Lohm J, Grahn M, Andersen Ø, von Schantz T (2001). Association between major histocompatibility complex class IIB alleles and resistance to Aeromonas salmonicida in Atlantic salmon.. Proc Biol Sci.

[28] Hoover GJ, el-Mowafi A, Simko E, Kocal TE, Ferguson HW (1998). Plasma proteins of rainbow trout (Oncorhynchus mykiss) isolated by binding to lipopolysaccharide from Aeromonas salmonicida.. Comp Biochem Physiol B Biochem Mol Biol.

[29] Ottinger CA, Johnson SC, Ewart KV, Brown LL, Ross NW (1999). Enhancement of anti-Aeromonas salmonicida activity in Atlantic salmon (Salmo salar) macrophages by a mannose-binding lectin.. Comp Biochem Physiol C Pharmacol Toxicol Endocrinol.

[30] Hoover GJ, Simko E, El-Mowafi A, Ferguson HG, Hayes MA (1998). Lipopolysaccharide-binding lectins and pentraxins in plasma of salmonids genetically resistant to Aeromonas salmonicida.. Faseb Journal.

[31] Smith VJ, Fernandes JM, Jones SJ, Kemp GD, Tatner MF (2000). Antibacterial proteins in rainbow trout, Oncorhynchus mykiss.. Fish Shellfish Immunol.

[32] Magnadottir B, Gudmundsdottir S, Gudmundsdottir BK (1995). Study of the humoral response of Atlantic salmon (Salmo salar L.), naturally infected with Aeromonas salmonicida ssp. achromogenes.. Vet Immunol Immunopathol.

[33] Griffin BR (1983). Opsonic effect of rainbow trout (Salmo gairdneri) antibody on phagocytosis of Yersinia ruckeri by trout leukocytes.. Dev Comp Immunol.

[34] Michel C, Gonzalez R, Avrameas S (1990). Opsonizing Properties of Natural Antibodies of Rainbow-Trout, Oncorhynchus-Mykiss (Walbaum).. Journal of Fish Biology.

[35] Cipriano RC (1983). Resistance of Salmonids to Aeromonas-Salmonicida - Relation between Agglutinins and Neutralizing Activities.. Trans Am Fish Soc.

[36] Freedman SJ (1991). The Role of Alpha 2-Macroglobulin in Furunculosis - a Comparison of Rainbow-Trout and Brook Trout.. Comparative Biochemistry and Physiology B-Biochemistry & Molecular Biology.

[37] Lee KK, Ellis AE (1991). Interactions between Salmonid Serum Components and the Extracellular Hemolytic Toxin of Aeromonas-Salmonicida.. Diseases of Aquatic Organisms.

[38] Huntly PJ, Coleman G, Munro ALS (1988). A Comparative-Study of the Sera of Brown Trout (Salmo-Trutta) and Atlantic Salmon (Salmo-Salar L) by Western Blotting against Aeromonas-Salmonicida Exoproteins.. Biochemical Society Transactions.

[39] Lindenstrom T, Sigh J, Dalgaard MB, Buchmann K (2006). Skin expression of IL-1beta in East Atlantic salmon, Salmo salar L., highly susceptible to Gyrodactylus salaris infection is enhanced compared to a low susceptibility Baltic stock.. J Fish Dis.

[40] Buchmann K, Uldal A (1997). *Gyrodactylus derjavini* infections in four salmonids: Comparative host susceptibility and site selection of parasites.. Dis Aquat Organ.

[41] Sakai M, Atsuta S, Kobayashi M (1991). Susceptibility of 5 Salmonid Fishes to Renibacterium-Salmoninarum.. Fish Pathology.

[42] Bruno DW (1986). Histopathology of Bacterial Kidney-Disease in Laboratory Infected Rainbow-Trout, Salmo-Gairdneri Richardson, and Atlantic Salmon, Salmo-Salar L, with Reference to Naturally Infected Fish.. Journal of Fish Diseases.

[43] Rolland JB, Winton JR (2003). Relative resistance of Pacific salmon to infectious salmon anaemia virus.. Journal of Fish Diseases.

[44] Gjedrem T, Gjoen HM (1995). Genetic variation in susceptibility of Atlantic salmon, Salmo salar L., to furunculosis, BKD, and cold water vibriosis.. Aquaculture Research.

[45] McIntosh D, Cunningham M, Ji B, Fekete FA, Parry EM (2008). Transferable, multiple antibiotic and mercury resistance in Atlantic Canadian isolates of Aeromonas salmonicida subsp. salmonicida is associated with carriage of an IncA/C plasmid similar to the Salmonella enterica plasmid pSN254.. J Antimicrob Chemother.

[46] Miller RA, Reimschuessel R (2006). Epidemiologic cutoff values for antimicrobial agents against Aeromonas salmonicida isolates determined by frequency distributions of minimal inhibitory concentration and diameter of zone of inhibition data.. Am J Vet Res.

[47] Buchmann K, Dalsgaard I, Nielsen ME, Pedersen K, Uldal A (1997). Vaccination improves survival of Baltic salmon (*Salmo salar*) smolts in delayed release sea ranching (net-pen period).. Aquaculture.

[48] Buchmann K, Larsen JL, Therkildsen B (2001). Improved recapture rate of vaccinated sea-ranched Atlantic salmon, *Salmo salar* L.. J Fish Dis.

[49] Gjedrem T, Gunnes K (1978). Comparison of Growth-Rate in Atlantic Salmon, Pink Salmon, Arctic Char, Sea Trout and Rainbow-Trout under Norwegian Farming Conditions.. Aquaculture.

[50] Dalsgaard I, Madsen L (2000). Bacterial pathogens in rainbow trout Oncorhynchus mykiss reared at Danish freshwater farms.. J Fish Dis.

[51] Ødegård J, Olesen I, Gjerde B, Klemetsdal G (2006). Evaluation of statistical models for genetic analysis of challenge test data on furunculosis resistance in Atlantic salmon (Salmo salar): Prediction of field survival.. Aquaculture.

